# Wearable Ultrafine Particle and Noise Monitoring Sensors Jointly Measure Personal Co-Exposures in a Pediatric Population

**DOI:** 10.3390/ijerph16030308

**Published:** 2019-01-23

**Authors:** Douglas Leaffer, Christopher Wolfe, Steve Doroff, David Gute, Grace Wang, Patrick Ryan

**Affiliations:** 1 Civil & Environmental Engineering, Tufts University, Medford, MA 02155, USA; David.Gute@tufts.edu; 2 Cincinnati Children’s Hospital Medical Center, Cincinnati, OH 45267, USA; Chris.Wolfe@cchmc.org (C.W.); Patrick.Ryan@cchmc.org (P.R.); 3 CD Business Group, LLC, Wenham, MA 01984, USA; Steve.Doroff@gmail.com; 4 Department of Public Health and Community Medicine, Tufts University School of Medicine, Boston, MA 02111, USA; grace.wang604109@tufts.edu

**Keywords:** noise, decibels, personal exposure measurement, exposure assessment, sensor technology, diesel emissions, particle number concentration, PNC, ultrafine particles, UFP, TPN

## Abstract

Epidemiological studies have linked both traffic-related air pollution (TRAP) and noise to adverse health outcomes, including increased blood pressure, myocardial infarction, and respiratory health. The high correlation between these environmental exposures and their measurement challenges have constrained research on how simultaneous exposure to TRAP and traffic noise interact and possibly enhance each other’s effect. The objective of this study was to deploy two novel personal sensors for measuring ultrafine particles (UFP, <100 nm diameter) and noise to concurrently monitor real-time exposures. Personal UFP monitors (PUFP, Enmont, LLC) were paired with NEATVIBEwear™ (Noise Exposure, Activity-Time and Vibration wearable), a personal noise monitoring device developed by the authors (Douglas Leaffer, Steve Doroff). A field-test of PUFP monitors co-deployed with NEATVIBEwear logged UFP, noise and ambient temperature exposure levels at 1-s resolution in an adolescent population in Cincinnati, OH to measure real-time exposures in microenvironments (transit, home, school). Preliminary results show that the concurrent measurement of noise exposures with UFP is feasible in a sample of physically active adolescent participants. Personal measurements of UFP and noise, measured prospectively in future studies, will enable researchers to investigate the independent and/or joint-effects of these health-relevant environmental exposures.

## 1. Introduction

Air pollution from traffic exhaust emissions and chronic ambient noise exposure frequently occur together in urban areas, adversely impacting population health outcomes. Epidemiological studies have linked both air pollution and noise to common adverse health outcomes such as increased blood pressure and myocardial infarction [1], and some research has shown adverse effects on pulmonary function due to the combined effects of air pollution and noise stress [2].

Most of the evidence supporting the link between acute and chronic exposures to air pollution and increased hospitalizations and mortality from cardio-pulmonary diseases has been based on epidemiological studies of human exposure to fine particulate matter (PM_2.5_). Recent studies, including the Tufts University-led Community Assessment of Freeway Exposure and Health Study (CAFEH) have focused on understanding the role of ultrafine particles (UFP) on health effects as a public health priority [3,4]. UFPs are defined as particles that are 100 nanometers or less in diameter (≤100 nm). Given their small size, UFPs contribute little to the mass of particulate matter in ambient air but are the dominant contributors to total particle number (TPN). In single pollutant models, UFPs were associated with incident wheezing, current asthma, lower spirometric values, and increased asthma-related emergency department visits among children [5]. Motor vehicles, especially those powered by diesel engines, have often been cited as a leading source of ambient UFP emissions and of deleterious effects on human health [6].

Traffic noise has been independently associated with various adverse health outcomes, most notably cardiovascular morbidity and mortality, including hypertension and ischemic heart disease [7,8]. Traffic noise has additionally been associated with impaired neurocognitive development and function in children and adults [9,10], reproductive failure and low birthweight [11], and diabetes mellitus as a possible metabolic outcome [12]; all of these outcomes are linked to exposure to air pollution as well. Research on noise exposure to urban cyclists in Belgium has shown that engine-related traffic noise encountered along the bicyclist’s route is a valid indicator of black carbon (BC), yet BC does not correlate with noise expressed as A-weighted equivalent sound pressure levels (LAeq) [13]. The Belgian study suggests utilizing simultaneous measurements of joint noise and TRAP (BC and UFP) to identify such contrasts in exposures [14]. 

A-weighting of sound pressure levels is based on early work by Fletcher and Munson [15] to establish equal-loudness curves dependent on both amplitude and frequency of sound. The A-weighted sound level metric has been found to correlate well with human perception of environmental noise and is specified for sound level meters (SLM) currently used for transportation and community noise studies [16]. Current transportation noise impact assessments are usually based on broadband A-weighted noise indicator, although many studies have shown that A-weighting can underestimate the important role low frequency noise (LFN) plays in loudness perception, annoyance, and speech intelligibility. Despite this, A-weighted sound pressure levels (SPL), or dBA has continued to be the predominant measurement descriptor for noise assessment [17]. Predictive models have been developed to estimate measured TPN adjusted for meteorological conditions for specific noise indicators (L_125Hz_ − L_2kHz_ = low to med frequency noise bands) [18]. A key constraint in the development and refinement of such models is the difficulty in capturing noise frequencies that are representative of traffic sources, rather than reliance on the conventional A-weighted SPL, dBA for model construction. Measuring the spectral content of the noise exposure is necessary to achieve valid spatiotemporal models [13].

Accurately measuring personal or breathing zone exposure to air pollutants remains a significant challenge to determining their impact on human health. The need for more precise exposure assessment is particularly evident for children and adolescents whose exposure can vary widely based on their time-activity patterns, including time spent outdoors, at home, school, and in vehicles. Of particular interest is exposure assessment of microenvironments where both UFP and noise exposures are, a priori, expected to be elevated—for example, in vehicles, during school commutes. This objective of this pilot field study was to jointly measure both UFP and noise exposures on a personal-scale, defined as a distance of <30 cm between the sampler and the exposure point (e.g., breathing zone, ear canals) [19], using wearable monitors for both constituents. 

## 2. Materials and Methods 

**Figure 1:**
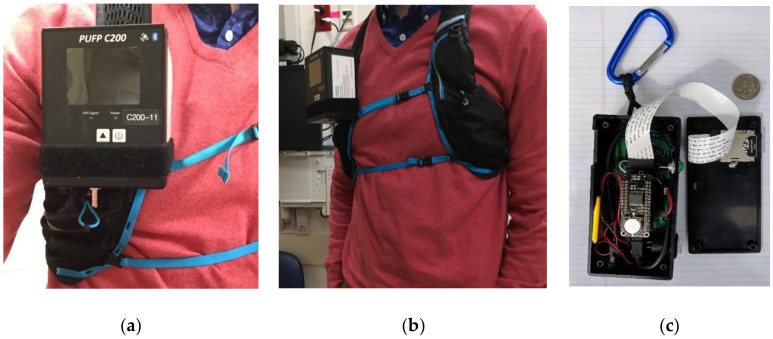
Photographs of PUFP C200 sampler and a NEATVIBEwear personal noise exposure monitor: (**a**) and (**b**) PUFP C200 Personal UFP particle counter worn by participant; (**c**) NEATVIBEwear noise monitor internal components. The dimensions of NEATVIBEwear are 13 × 7 × 4 cm (U.S. quarter shown for scale). NEATVIBEwear is worn by participants in the mesh pocket of the left shoulder strap (**b**). PUFP: Personal UFP.

To characterize personal exposures to both UFP and noise, we utilized two tools, both developed and validated in laboratory settings and capable of measuring personal-scale exposures to UFP and noise with high spatiotemporal resolution. The UFP monitoring device is a Personal UFP (PUFP, Enmont, LLC, Cincinnati, OH, USA) particle counter, model C200 (Figure 1a,b). The PUFP C200 is a portable, water-based condensation particle counter, with particle size ranging from 6 nm to ≥3 um, particle counting ranging from 0 to 2.0 × 10 ^5^ particles/cm^3^, counting accuracy ±10%, response time <0.5 s and sampling frequency of 0.1–1.0 s. The PUFP sampler operates on a Li-Po battery with 3 h of continuous operation and logs particle concentration, local time (Greenwich Mean Time, GMT). In addition, the PUFP incorporates a GPS receiver, which appends geolocation to corresponding UFP measurements. All data is recorded to an external micro SD card. The device dimensions are 7 × 10 × 13 cm and weighs 0.75 kg. Results of an initial field test, prior to this study, found the sensor to be mobile, rugged, and able to provide accurate spatiotemporal measurements of personal UFP exposure [20]. 

We paired the PUFP sampler with NEATVIBEwear™ (Noise Exposure, Activity-Time and Vibration wearable), a personal noise monitoring device developed by the authors (Douglas Leaffer, Steve Doroff) that allows users to view their noise exposure levels (A-weighted decibels, dBA) and monitor time-weighted exposures (Figure 1c). 

NEATVIBEwear architecture integrates an AdaFruit® (New York, NY, USA) Feather M0 AdaLogger with an ARM Cortex M0 processor, AdaFruit DS3231 I^2^C-integrated real-time clock (RTC) and Bluetooth Low Energy (BLE) messages to the application running on an Android mobile device. The dimensions of NEATVIBEwear are 13 cm × 7 cm × 4 cm, and the device weighs 0.21 kg. NEATVIBEwear includes an on-board SD card for data capture and is optionally integrated to a smartphone to allow for easy visual access to noise exposure levels, which are also displayed on an integrated OLED 128 × 32 I^2^C display. NEATVIBEwear incorporates an SPH0645 I2S built-in digital microphone to detect sound intensity of the microenvironment. The microphone has a working frequency range of 50 Hz to 15 kHz, a Signal-to-Noise Ratio of 65 dBA and Sensitivity of −29 to −23 dBFS (decibels relative to full scale), with a digital output (voltage) mapped to an instrument detection range of 50 to 110 dBA via an algorithm coded in C/C++ programming language. It should be noted that PUFP samplers generate an internal motor and pump noise of 48 dB, which is at the lower detection limit of NEATVIBEwear.

**Figure 2:**
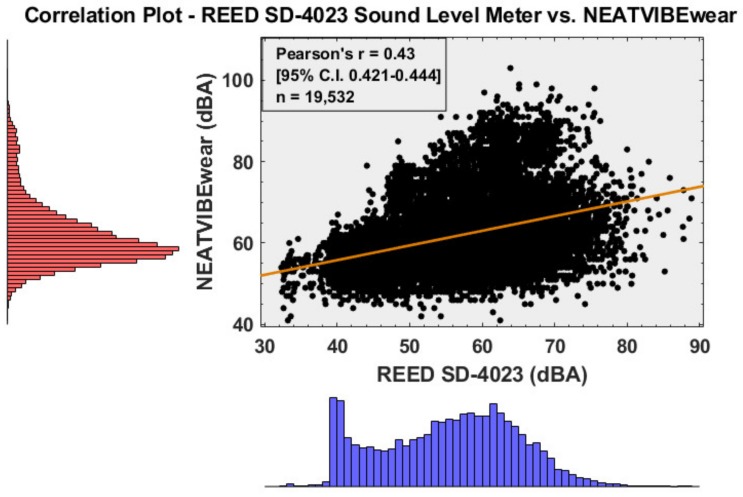
Correlation plot NEATVIBEwear vs. REED SD-4023 sound level meters.

The NEATVIBEwear device was calibrated to a REED instruments model SD-4023, class 2 commercial grade SLM with a sound level generator at 94 dB and 114 dB, both at a frequency of 1 kHz. A correlation plot of NEATVIBEwear vs. REED SD-4023 is shown in Figure 2, which indicates that the calibrated devices are moderately correlated (Pearson’s *r* = 0.43). The correlation is based on a co-location test in which both devices monitored ambient noise in several microenvironments: in-transit (car), college classroom, and a quiet office at 1-s intervals (*n* = 19,532). The NEATVIBEwear internal battery is rechargeable via micro USB port and provides a functional run time of 20 h per charge. NEATVIBEwear additionally incorporates an internal thermistor temperature sensor with a primary function to track temperature for calibration of the device’s DS3231 Real Time Clock (RTC). The digital temperature sensor provides an accurate measurement (~ ±5 degrees F) of ambient microenvironmental temperature. 

PUFP and NEATVIBEwear samplers were field-deployed in February 2018 on a subset of adolescent participants in Cincinnati, OH enrolled in the Ecological Momentary Assessment and Personal Particle Exposure (EcoMAPPE) Study. The study protocol was approved by the Cincinnati Children’s Hospital Medical Center (CCHMC) Institutional Review Board (IRB), and all participants provided signed consent (caregivers) and assent (adolescents) prior to study participation. Briefly, participants in the EcoMAPPE Study (ages 13–17) completed two personal sampling sessions of seven consecutive days each (14 total personal sampling periods/participant): one session during summer months and one session in winter. On each sampling day, participants wore the PUFP C200 for ~3 h. Participants are instructed to operate the sensor and measure exposures at different times throughout the sampling period and in different environments (i.e., while at school, in transit, etc.). In addition to the PUFP C200, EcoMAPPE participants received a study smartphone to link other sensors and apps including the PiLR Health Ecological Momentary Assessment (EMA) app. The PiLR EMA app prompts participants to answer brief questionnaires throughout the day based on time, location (e.g., proximity to home, school), and the smartphone’s accelerometer. 

Microenvironmental UFP exposures are assigned based on the speed and distance from participant specified locations: home (100 m), schools (400 m), and other (i.e., work; 400 m) locations. UFP concentrations measured outside of these locations exhibiting accelerometer speeds greater than 2 m/s are categorized as occurring while in transit. Measurements that are not categorized based on location and exhibit speeds <2 m/s are assigned to ‘other.’ Participants also record locations where they perceive high air pollution to be present and may upload images to give a visual depiction of their current environment. GPS data was also logged for the entirety of the sampling period using the madresGPS app; a Fitbit Charge 2 (San Francisco, CA, USA) to monitor heart rate, sleep quality, and activity; and a Spirobank Smart (MIR Group, Rome, Italy) to assess lung function. For the present analyses, a subset of EcoMAPPE participants was randomly selected to additionally wear the NEATVIBEwear monitor. The objective of this analysis is to demonstrate the usability of the NEATVIBEwear device and examine the correlation between personal noise and UFP measurements. Microenvironmental temperature exposures were also measured by NEATVIBEwear as an additional parameter of interest.

## 3. Results

### 3.1. Personal-Scale, Real-Time Monitoring of Microenvironmental UFP Exposures

**Figure 3:**
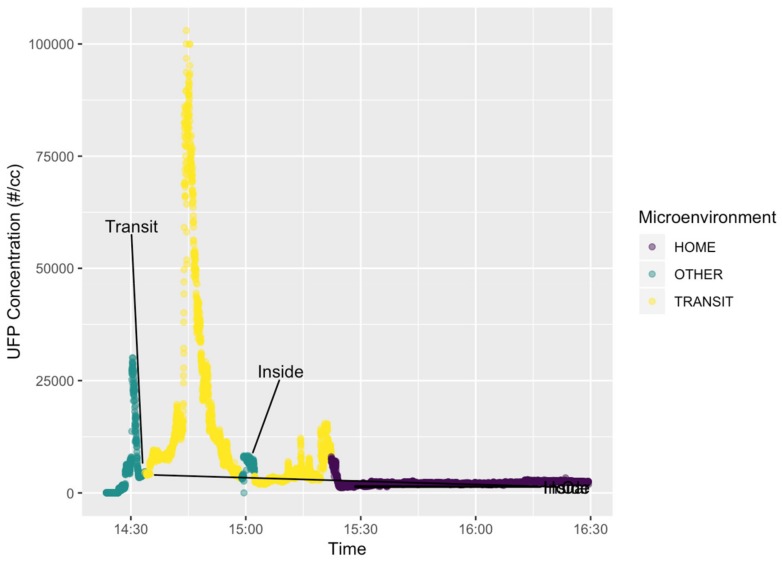
Plot of UFP exposure, colored by microenvironment, participant # 300012 (28 February 2018).

A field test of the PUFP C200 sampler and NEATVIBEwear noise monitor was conducted at the beginning of the joint deployment period (February 2018) for seven study participants. Two illustrative cases are presented here as examples of co-exposures to both UFP and noise, measured on a personal scale. Participant #300012, an adolescent female, age 13 at the time of participation in the study, she wore both personal exposure monitors on 28 February 2018, a routine mid-winter, mid-week school day. To monitor and assess real-time UFP concentrations with associated changes in microenvironments, we plotted a time-series plot of UFP (measured as PNC (# particles/cc)) for the 28 February 2018 sampling date, colored and annotated by microenvironment (i.e., while in transit, at home, other; Figure 3). 

### 3.2. Data Visualization of Concurrent UFP and Noise Personal Exposure Levels

**Figure 4:**
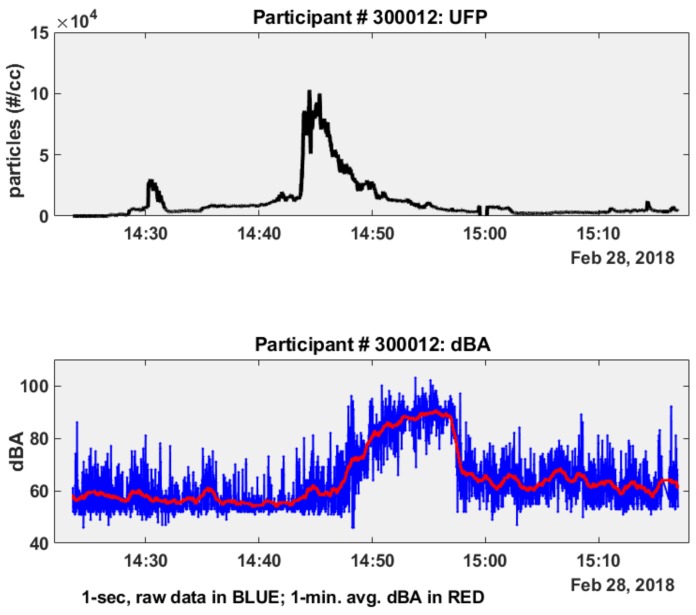
Time-series subplots of PNC (#/cc) and Noise (dBA), participant # 300012—28 February 2018.

To better visualize and understand the relationship between personal exposure to UFP and concurrent noise exposure from transportation sources, we plotted time-series subplots of the participant’s 1-s continuous exposure levels to both UFP and noise (dBA), with 1 min average dBA also plotted with a moving mean data smoothing function (MATLAB R2018a), presented in Figure 4. 

Joint data collection for participant # 300012 from 14:25 to 15:15HRS US Eastern Standard Time (EST) shows an increase in both UFP and noise exposures above baseline readings beginning at approximately 14:45HRS, while the participant was in transit (Figure 3). PNC levels (Figure 4, upper plot) increased above a baseline mean of 32.7 particles/cc (baseline standard dev. = 5.8; baseline median = 33 particles/cc) recorded during the first 3 min of the monitoring period to a maximum of 103,000 particles/cc at peak exposure at approximately 14:45HRS. Following peak UFP exposure, particle concentrations decreased during the final 10 min of the monitoring period (15:05 to 15:15HRS) to a post peak exposure mean of 3634 particles/cc (post-peak standard dev. = 1338; post-peak median = 3210 particles/cc), fully 2 orders of magnitude higher than the initial baseline UFP concentrations.

Concurrent noise exposures logged by NEATVIBEwear indicate that noise levels also increased during the transit (car) microenvironment for participant # 300012. NEATVIBEwear measured and recorded noise exposure levels with an in-transit mean of 51.8 dBA (in-transit standard dev. = 5.4; in-transit median = 49 dBA) during a 20-min baseline period prior to the UFP peak, escalating gradually during and following the UFP exposure to a maximum of 98 dBA (Figure 4, lower plot). Noise levels returned to a secondary baseline with a post-peak mean of 58.2 dBA (post-peak standard dev. = 6.7; post-peak median = 58 dBA) during the final 17 min of the sampling period at 14:58 to 15:15HRS (Figure 3, lower plot). Descriptive statistics for participant # 300012 for the full data set of UFP exposure (PNC, particles #/cc) and noise exposure (dBA) are presented in Table 1.

**Figure 5:**
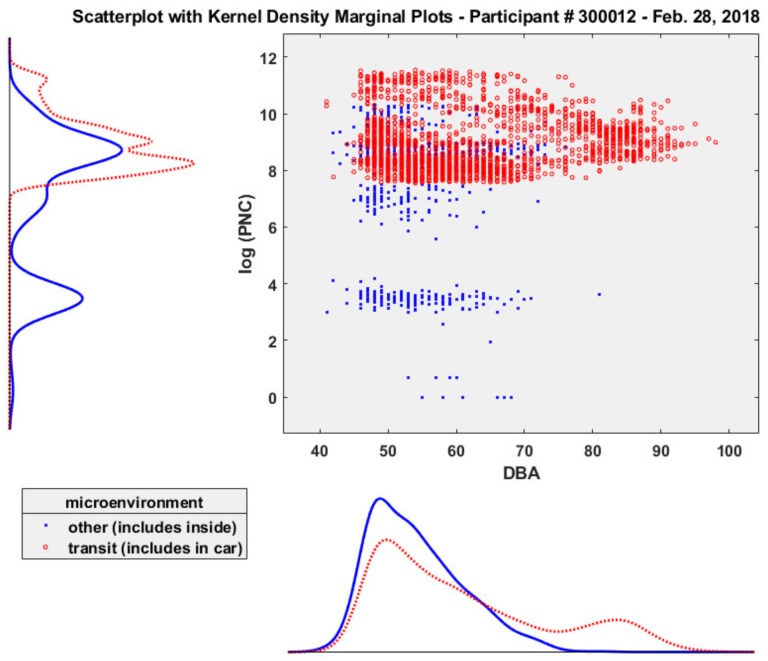
Scatter plot and kernel density plots of log PNC and noise, participant # 300012 (28 February 2018).

Figure 5 presents a scatter plot with marginal kernel density plots for log_(*e*)_ PNC and noise exposure grouped by microenvironment (transit, other) for participant # 300012. The scatter plot suggests that both the highest UFP concentrations and dBA levels which participant # 300012 was exposed to occurred during transit (including in car) (PNC > 30,000 particles/cc; dBA > 80). The lowest UFP concentrations (<3000 particles #/cc) and noise levels (<80 dBA) occurred while the participant was in other microenvironments (including inside). Given that both PNC and noise (dBA) are positively skewed, non-normal distributions (PNC skewness = 2.92; kurtosis = 11.5; dBA skewness 1.04, kurtosis = 3.11; *n* = 3169) (Table 1), we plotted a kernel distribution (Figure 5) since a parametric distribution cannot properly describe the data, and avoided making assumptions about the distribution of the data.

### 3.3. Heart Rate Measurements in Participants Exposed to UFP, Noise and Microenvironmental Temperature

**Figure 6:**
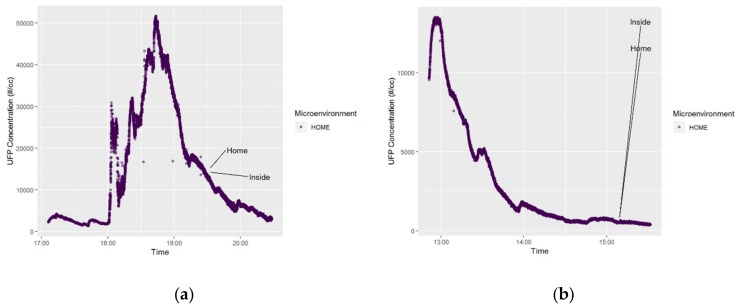
Plots of UFP exposure by microenvironment, participant # 300041 (21–22 June 2018).

Participants # 300012 and # 300041 were instructed to operate both the PUFP C200 and NEATVIBEwear sensors and measure exposures at different times throughout the sampling period and in different environments (i.e., while at school, in transit, etc.). A Fitbit Charge 2 (was additionally distributed to both participants to monitor heart rate, sleep quality, and activity. Participant # 300012 did not wear the Fitbit during the selected monitoring date (28 February 2018). No heart-rate data was recorded for this participant for this analysis. Participant # 300041, an adolescent male, age 16 at the time of participation in the study, wore the Fitbit on two consecutive days (21–22 June 2018). Figure 6 displays time-series plots for adolescent participant # 300041 illustrating the microenvironment in which the UFP exposures occurred. For this participant, all of the UFP exposures occurred while the participant was inside (at home), on both sampling days. While not transportation-related exposures, the time-series plots and data analysis for participant # 300041 are presented herein as illustrative examples of a home-bound participant’s personal exposure regimes in comparison to the transportation-source personal exposure of participant # 300012. 

**Figure 7:**
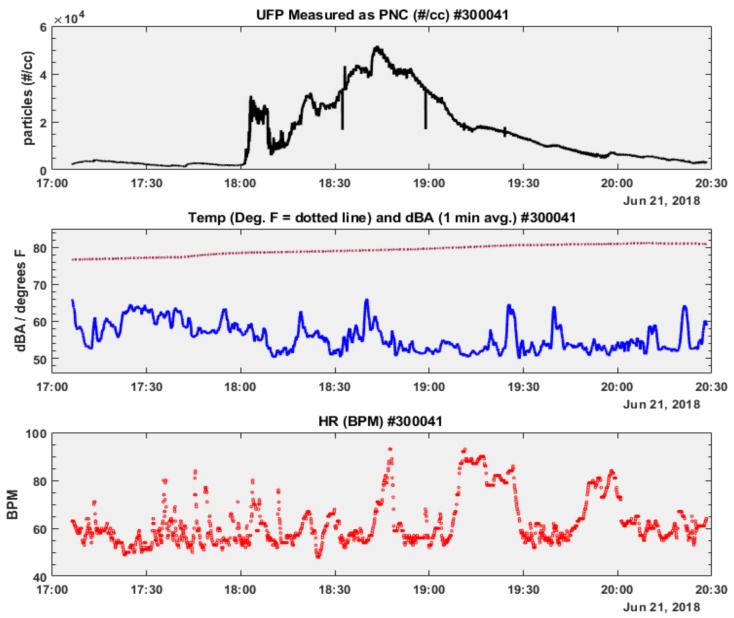
Time-series subplots of PNC, Temp., dBA, Heart Rate: participant # 300041 (21 June 2018).

**Figure 8:**
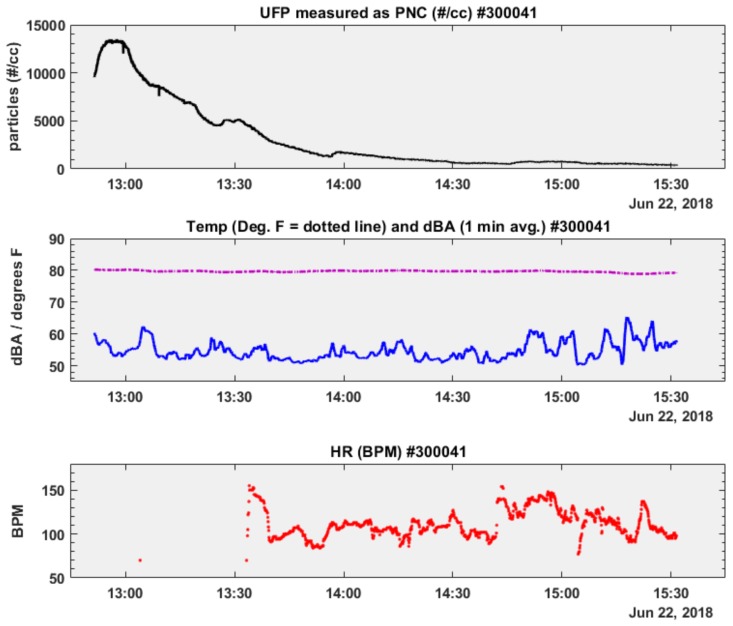
Time-series subplots of PNC, Temp., dBA, Heart Rate: participant # 300041 (22 June 2018).

To assess and evaluate real-time microenvironmental exposures and their effects on heart-rate, we plotted time-series plots of UFP and dBA with temperature as an added parameter of interest. Figure 7 and Figure 8 present these time-series plots for adolescent participant # 300041 during two consecutive early summer weekdays, 21 and 22 June 2018. Both sampling days recorded an elevated UFP exposure period occurring for approximately one hour, followed by an extended decreasing exposure period of similar or longer duration (PNC, particles #/cc, Figure 7 and Figure 8 upper plots). Compared to the transportation-related UFP exposures of participant # 300012, indoor (at home) UFP exposures in participant # 300041 were between 50–85% lower. Noise exposures (dBA, Figure 7 and Figure 8 middle plots) were within a narrow interquartile range (IQR) (21 June: IQR = 8 dBA; 22 June: IQR = 6 dBA) and showed little relative variability between sampling dates (21 June: CV = 0.11; 22 June: CV = 0.09, Table 2). Microenvironmental temperature exposures recorded by the a NEATVIBEwear internal thermistor are plotted as dotted lines in the middle subplots in Figure 7 and Figure 8. Given the indoor exposure regimes, microenvironmental temperature readings were limited to a narrow range (Table 2). 

Descriptive statistics for participant # 300041 UFP exposure (PNC, particles #/cc), noise exposure (dBA), microenvironmental temperature (degrees F), and heart rate (HR, beats per minute, BPM) are displayed in Table 2. 

**Figure 9:**
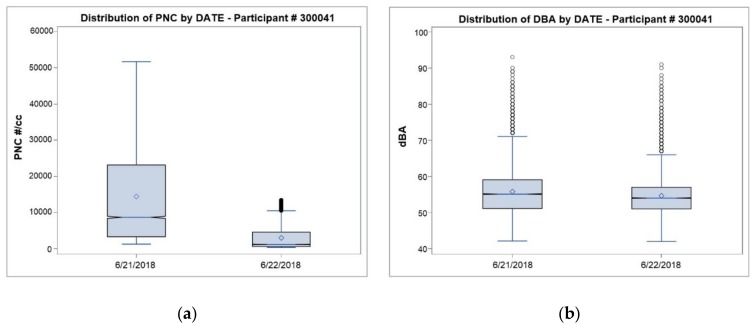
Boxplots of UFP (# particles/cc) and Noise (dBA), participant #300041 (21–22 June 2018).

**Figure 10:**
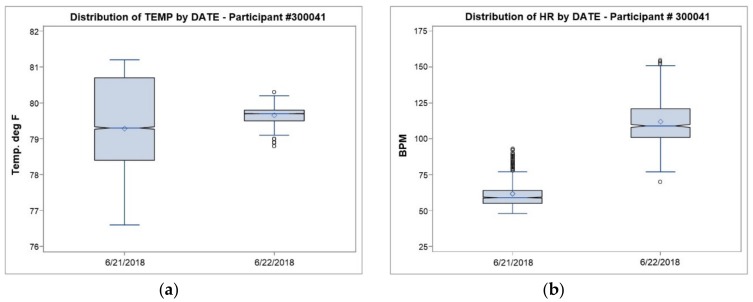
Boxplots of Temp. (^o^F) and Heart-Rate (BPM), participant #300041 (21–22 June 2018).

Heart-rate was measured during both sampling days (21–22 June 2018) in participant # 300041, who wore the Fitbit device. While there was little relative heart-rate variability between monitoring dates (21 June: CV = 0.15; 22 June: CV = 0.14, Table 2), the participant’s heart-rate was elevated on 22 June, although heart-rate data from 13:04 to 13:33 HRS (during the decline of the UFP peak) are missing. For a visual comparison of how the distribution of each covariate (UFP, noise microenvironmental temperature) varies by sampling day with the outcome variable (heart-rate), we plotted notched boxplots of each personal exposure parameter below in Figure 9 and Figure 10. 

## 4. Discussion

We found that personal-scale, microenvironmental exposure measurement with novel, wearable sensors is feasible for assessment and evaluation of co-exposures of UFP and noise on health outcomes (heart-rate). While participants in this field study proved to be cooperative in wearing the sensors, their compliance with study protocols was intermittent and, to some extent, beyond the control of the researchers. However, the data collected in this field study has proven to be valuable for protocol refinement in the remainder of the study (end date = February 2020) and will inform future personal-scale exposure assessment studies of similar design. We also found that measurement of real-time microenvironmental temperature using a wearable sensor provides an accurate measurement (~ ±5 degrees F) of ambient microenvironmental temperature, which is useful for understanding UFP and temperature collinearity. Most exposure studies utilize average daily temperature values from fixed site meteorological records as a proxy for actual exposures, which may vary considerably as participants move between microenvironments [21]. Transportation-related air pollution (TRAP) was measured successfully here in real time using a PUFP C200 wearable sampler as particle number concentration (# particles/cc). This serves as a proxy for UFP exposure. UFP measurements were collected concurrently with noise exposure data (dBA) by NEATVIBEwear, a wearable noise monitoring device. 

### 4.1. Interpretation of Measurements of Participants Exposed to UFP and Noise 

Field study data from adolescent participant # 300012, who reported their exposure categories as “transit” or “car” and “inside” or “home” have illustrated the value and relative convenience of using wearable sensors for personal-scale monitoring of the co-exposures of UFP and noise. Our results for participant # 300012 suggest that traffic-related UFP and transportation noise exposures can be measured in real time with current sensor technology. The UFP exposures plotted in Figure 3 (participant # 300012) are illustrative of a typical commute pattern, which, in this study, featured a commute from school followed by an inside or indoor exposure period. The recorded PNC measurements suggest an immediate and short-term exposure to UFP while the participant changed microenvironments (other to transit) (Figure 3). 

Concurrent noise exposures logged by NEATVIBEwear (Figure 4, lower subplot) indicate that noise levels also increased during the transit (car) microenvironment for participant # 300012. There was approximately a 10 min time-lag between peak UFP concentration (103,000 particles/cc) and peak noise exposure (98 dBA), sub-plotted in Figure 4. The elevated peak in UFP exposure might correlate with so-called “stop and go” traffic, particularly if the participant’s vehicle (and PUFP sampler) was a short distance to the exhaust tailpipe of the preceding vehicle. This distance and vehicle speed could potentially be established with the GPS and accelerometer data the study participants logged while in-transit. Another possible explanation for the UFP vs. noise time lag could be that participant #300012 may have travelled behind a high UFP-emitting, diesel vehicle previous to the peak in dBA and UFP exposures peaked at a period before the vehicle increased in acceleration. We have found previously that UFP concentrations may be higher at intersections due to increased acceleration of the vehicles. The gradually increasing and elevated noise levels (Figure 4) likely correlate with a higher speed portion of the vehicle trip. This is attributed to rolling tire noise and aerodynamic noise of the vehicle itself. At a higher speed, the rapid drop in UFP concentration (Figure 4) may be due to more efficient in-vehicle ventilation and longer distance to preceding vehicles [22].

Given these possible scenarios, the scatter plot of log UFP vs. noise presented in Figure 4 does not indicate a correlation between these variables for this participant but does suggest that both the highest UFP concentrations and dBA levels which participant # 300012 was exposed to occurred during transit and the lowest UFP concentrations and noise levels occurred while the participant was in other microenvironments (including inside). Establishing such a correlation is feasible in future field work using wearable sensors for personal-scale exposures across multiple participants in varying microenvironments. For more precise correlations of UFP and noise, analyzing the spectral content of the in-transit noise would be informative to verify if the low frequency content of the measurements correlates with the UFP exposure inside the vehicle. We believe that spectral analysis of selected noise peaks will demonstrate low frequency noise (250 Hz and below) to be correlated with higher UFP levels predominately from diesel exhaust emissions from large trucks and heavier gross weight vehicles, with lower UFP concentrations corresponding with high frequency noise (>2000 Hz) [23]. 

### 4.2. Challenges in Measuring Health-Based Outcomes in Personal-Scale Exposure Studies

Jointly measuring UFP and noise exposures on a personal-scale (<30 cm between the sampler and the exposure point—i.e., breathing zone, ear canals) using wearable monitors for both UFP and noise is a feasible approach. Integration of a Fitbit device to monitor heart rate, sleep quality, and activity, and a Spirobank Smart to assess lung function complements the other wearable instruments deployed here to form a suite or set of technologies to better understand possible relationships between UFP and noise exposures and health outcomes. While measurement of personal-scale UFP is still an emerging approach, the literature cites only one study that measured exposure and health effects in children using personal UFP monitors [24]. Integration of low-cost, accurate and precise noise monitoring devices into UFP monitoring studies remains an enticing possibility to disentangle the confounding presented by these simultaneous exposures. The rate limiting step to date has been mainly one of instrumentation and measurement techniques.

An important objective of this field study was to measure health-based outcomes in adolescent participants based on exposure to real-time UFP concentrations with both noise and changes in microenvironmental temperature exposure. Based on the quality of data presented herein, microenvironmental monitoring of UFP and noise with the paired PUFP sampler and NEATVIBEwear devices (Figure 1) presents a feasible approach to collecting more fine-grained and robust exposure data than other methods such as fixed UFP monitoring sites or noise proxies to predict UFP exposures. Furthermore, the data presentation illustrates the potential for opportunities as well as difficulties in collecting and evaluating personal-exposure, real-time monitoring data. Additionally, the data quality output from personal-scale monitoring sensors deployed in this study facilitates the ability to construct a regression-based model in which the covariates UFP, noise and microenvironmental temperature are the predictor variables and heart-rate as the outcome and is dependent on any of the predictors and/or their interaction. Such a model could be further stratified by both microenvironment of exposure and activity category. 

### 4.3. Advantages in Measuring Real-Time Noise Exposures Using Wearable Sensors

The traditional methods of conducting noise measurement studies involve a collection of noise samples manually, using professional sound level meters (SLMs) that must comply with national and international standards. The relative cost of high-grade SLMs and trained personnel required to calibrate and measure noise levels presents financial and logistical drawbacks [25]. The recent proliferation of smartphones, their constant network connectivity, the built-in GIS functionality, and numerous “apps” available for user interactivity present distinct advantages over unconnected and often bulky and expensive professional SLM instruments [26]. 

However, reliance on smartphones for sound level data collection in noise exposure studies presents its own drawbacks, such as the micro-electro-mechanical-system (MEMS) built-in microphones used in smartphones. MEMS microphones have certain limitations due to their miniature size and circuit board placement, which affect their dynamic range and signal-to-noise ratio response [27]. Another major constraint presented by the built-in microphones is the lack of access and inability to perform periodic or pre-measurement calibration. Additionally, smartphones are difficult to utilize as dedicated SLM devices in exposure studies due to the user/operator’s frequent needs to answer phone calls, send and receive texts messages, run applications and other uses which decrease battery life.

NEATVIBEwear was conceived, designed and engineered to be both a stand-alone wearable noise monitoring device of low cost, with functionality to log noise exposure data to an on-board SD card for data capture and is optionally integrated to a smartphone to allow for easy visual access to noise exposure levels. A study published in the Journal of the Acoustical Society of America (JASA) suggests that using external calibrated microphones greatly improves the overall accuracy and precision of smartphone sound measurements and removes much of the variability and limitations associated with the built-in smartphone microphones [26]. By integrating an external, calibrated microphone into the NEATVIBEwear device, with flexibility to re-calibrate the microphone unlike a smartphone, we anticipate the ability to obtain measurements within ±2 dB of a SLM reference. 

### 4.4. Limitations of This Study

A possible limitation in the use of NEATVIBEwear as a dedicated noise exposure monitoring device is the inability of the device to differentiate recorded noise patterns such as traffic noise and other sounds, including conversation, shouting, laughter or background music playing. This could potentially be addressed with a band-pass filter device that passes noise frequencies within a certain range and rejects (attenuates) frequencies outside that range, or via development of an algorithm to post-process the raw noise data to distinguish between environmental noise and the aforementioned interfering sounds. The latter approach was achieved with good results in a health study conducted in Peru in which low-cost wearable microphones were deployed on patients recovering from pulmonary tuberculosis (TB) to record and analyze coughing episodes and distinguish TB-related coughing from environmental and other background interfering noise [28].

NEATVIBEwear, in its current release, does not allow for 1/3-octave band or spectral measurements of traffic noise. This could be possible with an extended, re-engineered version of NEATVIBEwear in a follow-up (future) study or with a commercial-grade sound level meter. For validation of spatiotemporal UFP and noise models, measuring the spectral content of the noise exposure is necessary [13].

To our knowledge, this field program is the first study to integrate real-time, wearable sensors to jointly measure both UFP and noise exposure on a personal-scale with direct, real-time measurement of a health-based outcome (heart-rate) in study participants. Other strengths of this study include the ability to demonstrate direct measurement of microenvironmental temperature as a potential predictor, along with UFP and/or noise and their interactions, of heart-rate as a health-based outcome. This challenges the usual reliance on models of average daily temperature values from fixed site meteorological records as a proxy for actual exposures, which may vary considerably as participants move between microenvironments [21]. A further strength of the full prospective study, as it progresses, is the ability to network via the cloud multiple NEATVIBEwear devices worn by different participants simultaneously to produce a real-time noise exposure map of the study area and compare this with UFP readings for establishment of high exposure risk zones both spatially and temporally. 

## 5. Conclusions

The primary objective of this field study was to deploy two novel personal sensors for measuring ultrafine particles (UFP) and noise to jointly monitor real-time exposures in microenvironments. The study focused on evaluating the feasibility of simultaneously measuring personal exposures to UFPs and noise with focus on the quality of the data and its value for interpretation. A secondary objective of this analysis was to demonstrate the usability of the NEATVIBEwear device developed for this study as an easy to deploy tool for integration into ongoing and future exposure studies where noise exposure is a parameter of interest.

This pilot field study demonstrates that both UFP and noise were feasibly measured on a personal scale for adolescent participants in different and changing microenvironments. Accurately measuring personal exposure to air pollutants, particularly UFP, remains a critical limitation to understanding their impact on human health. This field study demonstrates the ability of new tools to accurately measure personal UFP exposure, noise and temperature with high spatiotemporal resolution. Such improvements in exposure assessment are likely to benefit future applications in epidemiologic studies.

## Figures and Tables

**Table 1:** Descriptive statistics for participant # 300012.

Feb 28, 2018 ^(1)^	PNC (#/cc)	Noise (dBA)
minimum	1	41
maximum	103000	98
mean	11765	59.3
median	5620	56
standard dev.	17342	11.8
cv ^(2)^	1.47	0.20
skewness	2.92	1.04
kurtosis	11.5	3.11

^(1)^ # observations, *n* = 3169; ^(2)^ cv = coefficient of variation. PNC: particle number concentration.

**Table 2:** Descriptive statistics for participant # 300041.

21 June 2018 ^(1)^	PNC (#/cc)	Noise (dBA)	Temp. (^o^F) ^(3)^	Heart Rate ^(4)^
minimum	1310	42	76.6	48
maximum	51700	93	81.2	93
mean	14444	55.7	79.3	61.7
median	8710	55	79.3	59
standard dev.	13572	5.9	1.4	9.3
cv	0.94	0.11	0.02	0.15
**22 June 2018 ^(2)^**	**PNC (#/cc)**	**Noise (dBA)**	**Temp. (^o^F) ^(3)^**	**Heart Rate ^(4)^**
minimum	354	42	78.8	70
maximum	13500	91	80.3	155
mean	3001	54.6	79.7	112
median	1200	54	79.7	109
standard dev.	3563	5.1	0.28	15.7
cv	1.2	0.09	0.004	0.14

^(1)^ # observations, *n* = 12127; ^(2)^ *n* = 9612; ^(3)^ NEATVIBEwear microenvironmental temperature; ^(4)^ BPM.
